# Losing out to improve group fitness

**DOI:** 10.7554/eLife.75243

**Published:** 2021-12-17

**Authors:** Jos Kramer, Rolf Kümmerli

**Affiliations:** 1 Department of Quantitative Biomedicine, University of Zurich Zurich Switzerland

**Keywords:** specialisation, synergy, cooperation, division of labor, None

## Abstract

A mathematical model provides clues as to why members of a group divide tasks between them even when specialisation reduces the performance of individuals.

**Related research article** Cooper GA, Frost H, Liu M, West SA. 2021. The evolution of division of labour in structured and unstructured groups. *eLife* **10**:e71968. doi: 10.7554/eLife.71968

In *The Wealth of Nations*, political economist Adam Smith stated that division of labour is favoured if specialisation leads to an “*increase of dexterity in every particular workman”* so that the same number of people show a *“great increase of the quantity of work"*. Simply speaking, individuals divide tasks between them when specialisation leads to accelerated returns for everybody involved (Bourke, 2011; Cooper and West, 2018; Michod, 2006). While this concept was initially devised for human societies, it turned out to be applicable to biological systems.

Examples of individuals within social groups specialising in different tasks are plentiful in nature and cover the entire range of biological complexity: from subgroups of bacteria producing different compounds of a biofilm (Dragoš et al., 2018), to ant queens specialising in egg laying and ant workers in brood care (Hölldobler and Wilson, 1990). This scientific concept is intuitive, easy to grasp and solves the challenge of how division of labour can evolve. But, does it cover all scenarios of how division of labour can arise? A recent study showed that in groups where individuals are organized into a particular spatial arrangement (also known as topology) and can only cooperate with direct neighbours, division of labour can evolve even when specialisation makes individuals less rather than more efficient (Yanni et al., 2020). New provoking hypotheses often spur controversy, and these findings are no exception. After all, to work harder but get less out of it seems, at first glance, not compatible with evolutionary theory.

Now, in eLife, Guy Cooper and colleagues from the University of Oxford – Hadleigh Frost, Ming Liu and Stuart West – report the results of a new mathematical model probing this hypothesis (Cooper et al., 2021). The model explored the conditions required for a population of cells, similar to the social groups reported by Rueffler et al., 2012, to divide reproduction and helping between group members. Each cell can differentiate into either a helper, which sacrifices its fecundity to increase the viability of other members, or a reproductive, which has higher fecundity but is less cooperative.

Cooper et al. found that reproductive division of labour can evolve when it improves the overall fitness of the group (Figure 1) and arises through three scenarios: (1) specialisation that results in accelerated returns for individuals; (2) physical characteristics that predispose some cells to become helpers and others to become reproductives; or (3) when specialisation is reciprocal and synergistic, meaning that by losing fitness helpers disproportionally benefit reproductives. The first scenario is the classic case, but how relevant are the other two, and how do they link to group topology?

**Figure 1.**
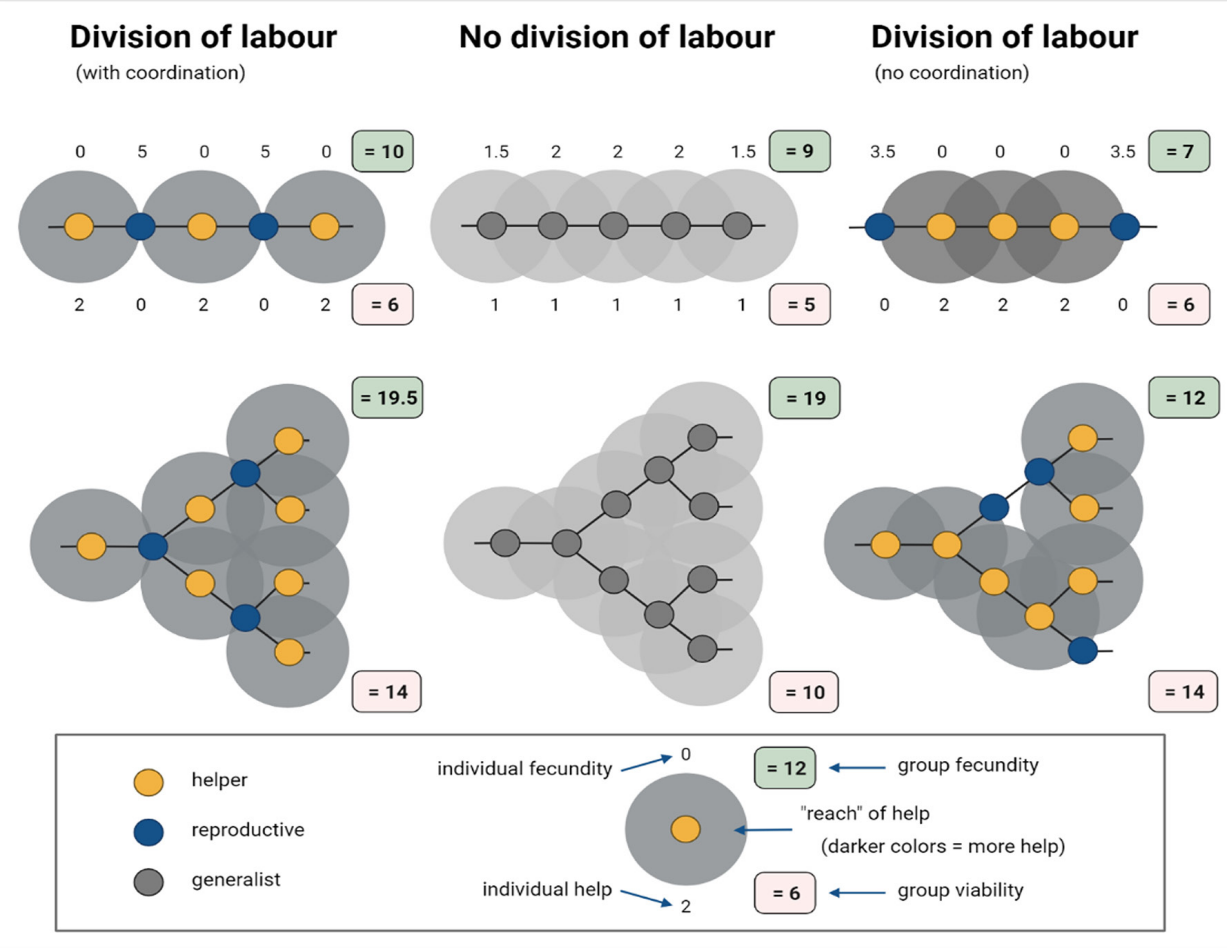
Simplified model of how division of labour evolves between cells in spatially organized groups. In the model created by Cooper et al. interactions occur either between helper (yellow) and reproductive (blue) cells, or among generalists (grey). Here, the representative model created for this figure assumes that generalists invest equally into help (*h* = 1) and personal fecundity (*f* = 1), whereas specialised cells invest fully into either helping (*h* = 2; *f* = 0) or fecundity (*f* = 2; *h* = 0). Among generalists (middle), the eventual fecundity of a cell (*F*) is increased through help from neighbours: in this example, *F = f + 0.5 n*, where *n* is the number of neighbours. Reproductives are more efficient than generalists in converting help to fecundity, and this synergistic benefit leads to a higher final fecundity: F = f + n*s, where *n* represents the number of neighbours and *s* represents the synergistic benefit*.* For example, when cells are arranged into a single column, also known as a filament structure (top), and their differentiation is coordinated (left), the fecundity of reproductives (*f = 2*), which are neighboured by two helpers, increases to five when *s* = 1.5; the fecundity and help values of individual cells are indicated above and below them, respectively. In filament structures, and also branched structures (bottom), this coordinated differentiation results in the total fecundity (green box) and viability (red box) of the group being higher than in populations that did not specialise and divide their labour (middle). However, when differentiation is not coordinated (right), division of labour decreases total group-level fecundity.

Image credit: Figure created using BioRender.com

Cooper et al. used their mathematical model to investigate three topologies: branching, when cells form tree-like structures; filaments, when cells form a single column; and well-mixed group structures, when each cell is connected to all other cells in the group. They found that division of labour can evolve in all three topologies even when specialisation leads to diminishing returns for individuals (that is, instances where the more support helpers provide the less efficient they are at helping). However, the underlying forces that drive this evolution differ between the three topologies.

In branching topologies, division of labour with diminishing returns arises from physical differences related to where cells are positioned in the group (scenario two): cells with fewer neighbours have reduced costs from refraining to reproduce and specializing into helpers. Conversely, in one-dimensional filaments, division of labour can evolve via reciprocal specialisation (scenario three) provided that the support given to reproductives increases their fecundity more than helpers have given up by specialising. Notably, this scenario also works in well-mixed groups, but only under the stringent assumption that helpers provide extreme levels of support. Altogether, these findings reveal that specific topological constraints are not required for reproductive division of labour to evolve, but allow it to happen under a larger range of conditions – that is, scenarios that differ in terms of the amount of help provided and how much it generates in return.

There is one important caveat with these results: the models assume that individuals can coordinate who differentiates into a reproductive or a helper cell. Without this coordination, division of labour turns out to be disastrous and reduces the overall reproductive fitness of the group (Figure 1; right). Cooper et al. note that this might limit the scope of spatial topology favouring division of labour in instances where returns for individuals are reduced. After all, coordination is more likely to evolve after division of labour has occurred and cells have already developed specific roles (Liu et al., 2021).

The insights by Cooper et al. are conclusive, novel and expand theoretical knowledge on how division of labour evolved. Nonetheless, empirical scientists might ask whether this is a purely theoretical exercise and whether there are real-world examples for scenarios involving non-accelerating returns. As this is currently unclear, Cooper et al. have provided guidelines for how to test for these scenarios in biological systems which will likely lead to examples in nature surfacing in the near future.
